# Role of Experiential Learning in the Desensitization of a Medical Student: Our Perspective

**DOI:** 10.31729/jnma.8164

**Published:** 2023-05-31

**Authors:** Simran Bista, Jyoti Chand

**Affiliations:** 1 Kathmandu Medical College and Teaching Hospital, Sinamangal, Kathmandu, Nepal

**Keywords:** *cadaver*, *emotions*, *experiential learning*, *medical students*

## Abstract

Being a medical student, it is really important to balance all sorts of emotions either positive or negative. Desensitization plays a significant role in shaping medical students' way to become efficient physicians. In this article, we discuss the effectiveness of experiential learning from the early medical student days such as in cadaveric dissection hall, operation theatre, and clinical rotation. The journey of desensitization among medical students is beneficial in situations that require emotional resilience over emotional instability. Experiential learning helps medical students in knowledge retention and a better understanding of their learning strengths and areas of improvement.

## INTRODUCTION

Desensitization is defined as the process of developing tolerance against negative stimuli. A journey of desensitization among medical students is beneficial in situations that require emotional resilience over emotional instability.^1^ Experiential learning is constructing knowledge and learning through deliberate practice. Whether it be the first encounter with a cadaver in the dissection hall or pricking one's finger and preparing a smear from one's own blood or first time entering into operation theatre (OT) or encountering dead bodies in forensic medicine. Medical students may experience a range of emotions, both positive and negative, such as interest, joy as well as melancholy, anxiety, and rage throughout their undergraduate years.^2^

## CADAVERIC DISSECTION EXPERIENCE

Going down memory lane cadaveric dissection was the first thing we encountered as medical students. Cadaver dissection is regarded as one of the most beneficial learning experiences in anatomy which utilizes the concept where the dead teaches living. First entering the dissection hall filled with preserved corpses and the characteristic smell of formalin evoke physical symptoms such as headache, and nausea, along with emotional reactions including anxiety, crying, and feeling guilty. Although cadaveric dissection is unique learning in itself, diverse learning experiences are sought by students in the same setting. Repeated encounter with dead bodies makes it less horrifying and with the help of professors and peers, it gradually desensitizes medical students to overcome fears compared to the initial encounter.

Anatomy which was initially found stressful after practicing dissection again and again till it develops an understanding of the human body gradually grasps the interest making it fun to read and understand. Practicing with the help of friends and professors helped us to cope with psychological reactions in the dissection hall. They must become actively involved in the dissection process to get used to it.

## OT EXPERIENCE

Medical students in their clinical years devote the majority of their time to learning from actual patients during their rotations in the medical ward and surgical theater. Although this experience is very helpful for aspiring doctors, it can also be very difficult to encounter real-clinical situations for the first time. The medical students' learning as well as their future career choices will be influenced by this experience. Most of the medical students described the first experience to be detrimental to negative emotions such as anxiety, fear of syncope, and shame. Medical students may become desensitized to the operating room after a period of familiarization, during which they become accustomed to the physical environment of the theater and are socially integrated by the surgeons.^3^

The first time we ever entered an operating room was during our surgery clinical rotation where we observed laparoscopic cholecystectomy. While entering the operation theatre we were not aware of proper attire for the operating room and we weren't supposed to touch anything inside the operation theatre. After being instructed, we changed into our scrubs and entered the operating theatre. The bright surgical lights over the bed and all the instruments that were positioned exactly where they could be reached during the surgery were the first things that caught our attention. From one side of the room, we watched the entire process, including what the surgeons were doing to the patient's body on the big screen. Even though we had studied the procedure in theory, I believe you could never psychologically prepare for it before actually seeing it done. Everyone involved in the procedure-including the anesthesiologist, nurses, and surgeons-was dressed in scrubs and concentrated on finishing the procedure while taking into account the patient's emotional connection. While we were observing the operation, a rollercoaster of emotions was flowing over our heads, and observing each other, some students were excited, some experienced near syncope experience and some had a somewhat neutral reaction. But on subsequent visits to the OT throughout a month-long clinical rotation, each visit got less intimidating. Although experiential learning during the surgical rotation can be difficult for medical students, an alternative can be practicing on suture kits and being observant during the procedure.

## OBSTETRICS AND GYNECOLOGY ROTATION

Medical students have the opportunity to experience a variety of obstetrics and gynecological procedures during the clinical rotation. For medical students, the obstetrics surgical rotation is a similarly intimidating experience as their first time in an operating room. Although it can be thrilling to see a woman give birth, the obstetrician's job is very stressful because of the physical setting, the emotions present, and the need to assure the child's safety without endangering the mother's health. While a drop of blood and fear of a needle were used to evoke negative emotions in medical students during physiology practical sessions in basic years, we could not possibly comprehend the blood loss during one delivery.

Kolb's learning cycle comprises four key steps of experiential learning i.e. concrete experience, reflective observation, abstract conceptualization, and active experimentation.^4^ Active experimentation might not be of relevance when it comes to rotation in the surgical ward but simulation training, which is a creation of true-to-life learning and mirrors real-life scenarios, can be beneficial to desensitize medical students to witness and potentially be actively involved in the clinical environment in the future.^5^

## WAY FORWARD

Learning from experience and discovery at the same time paves the way for desensitization to handle traumatic events and get through hardships. Experiential learning helps in transforming theoretical knowledge into practice and also preparedness of medical students for efficient physicians in the future. Effective desensitization along with experiential learning plays a significant role in increasing student understanding of the relevance of medical professionalism.
